# “It is good, but I can’t afford it …” potential barriers to adequate prenatal care among Afghan women in Iran: a qualitative study in South Tehran

**DOI:** 10.1186/s12884-020-02969-x

**Published:** 2020-05-06

**Authors:** Omid Dadras, Ziba Taghizade, Fateme Dadras, Leyla Alizade, Seyedahmad Seyedalinaghi, Masako Ono-Kihara, Masahiro Kihara, Takeo Nakayama

**Affiliations:** 1 Department of Health Informatics, Graduate School of Medicine, Kyoto University, Kyoto, Japan; 2 Nursing and Midwifery Care Research Center, Tehran University of Medical Sciences, Tehran, Iran; 3 Department of Obstetrics and Gynecology, Tehran University of Medical Sciences, Tehran, Iran; 4 Iranian Research Center for HIV/AIDS, Iranian Institute for Reduction of High-Risk Behaviors, Tehran University of Medical Sciences, Tehran, Iran; 5 Global Health Interdisciplinary Unit, Center for Promotion of Interdisciplinary Education and Research, Kyoto University, Kyoto, Japan; 6 Department of Health Informatics, Graduate School of Medicine, Kyoto University, Kyoto, Japan

**Keywords:** Prenatal care, Afghan women, Barrier, Iran

## Abstract

**Background:**

An estimated 96% of registered refugees in Iran are Afghan. Almost half of them are young women at the reproductive age. The adequate maternity care is crucial for healthy pregnancy. There is limited knowledge regarding the access and adequacy of maternity care among Afghan women in Iran. The reports from ministry of health (MOH) implicate higher prevalence of perinatal complications in Afghan population. This mainly attributed to the inadequate prenatal care during pregnancy. Therefore, this paper explores the potential barriers to prenatal care among Afghan women in Iran.

**Methods:**

Using convenience sampling, thirty pregnant Afghan women were recruited at three community health centers with the highest number of Afghan visitors in Tehran, the capital city of Iran. Data were collected through face-to-face interviews in Persian language using an interview guide. The interviewers were two bilingual Afghan graduate midwifery students. Each interview lasted for an hour. The questions regarding the concerns and experienced obstacles in seeking prenatal care were asked. The interviews were transcribed into original language (Persian) and analyzed using content analysis and further translated back into English. The main themes were extracted grouping the similar codes and categories after careful consideration and consensus between the researchers.

**Results:**

The financial constraints and lack of affordable health insurance with adequate coverage of prenatal care services, particularly the diagnostic and screening tests, were the most frequent reported obstacles by Afghan women. In addition, personnel behavior, transportation issues, stigma and discrimination, cultural concerns, legal and immigration issues were also mentioned as the source of disappointment and inadequate utilization of such services.

**Conclusions:**

The findings of present study emphasize the necessity of available and most importantly, affordable prenatal care for Afghan women in Iran. Providing an affordable health insurance with adequate coverage of prenatal and delivery services, could reduce the financial burden, facilitate the access, and ensure the maternal and child health in this vulnerable population. The issues of fear and concern of deportation must be removed for at least illegal Afghan mothers to ensure their access to maternity care and improve the health of both mother and offspring.

## Background

Iran is the second country with the highest number of Afghan refugees and immigrants during last decades [1]. Afghans consist almost 96% of registered refugees in Iran, accounting for 3% of total population in Iran [2]. One-third of this population reside in Tehran, the capital city [3]. The Afghan women are relatively young and approximately half of them are at childbearing age [1] with abundant reproductive needs. Therefore, adequate and affordable reproductive and maternity cares is essential to ensure the maternal and child health and reduce the adverse birth outcomes among this vulnerable population [4–6].

In Iran, as of 2016, all registered Afghans had become eligible to apply for the public health insurance. Thus, the insured Afghans could now receive the same health services, covered by public health insurance, as Iranian citizens [7]. Unfortunately, the situation for undocumented Afghans is not clear. Despite the available health insurance for Afghans which cover the basic maternity care, it appears that the majority of Afghan women do not have health insurance and overall utilization of such services is not adequate among them. On the other hand, some secondary and tertiary prenatal services such as screening tests and prenatal sonography are not covered by public health insurance in some institutions and hospitals [7]. The high cost of these procedures reduces the utilization of such services by Afghan women, particularly the undocumented ones, and pose them at the higher risk for adverse perinatal outcomes. In this regard, formulating strategies to facilitate the access of Afghan women to prenatal care and address their reproductive needs are critical. However, this requires a comprehensive knowledge and understanding of existent issues and potential obstacles in seeking prenatal care among Afghan pregnant women.

Although Afghan people are the largest refugee population in Iran, there is scarcity of research on their health needs and demands, particularly among female population. This is mainly because of the patriarchal approach in Afghan society in which the women’s needs are often ignored or missed [8]. Afghan women accessing public health services are compromised as they are often not allowed to appear in public without the permission of men in the family [3]. It is also hard to reach Afghan women in society. Therefore, the existent studies mainly involve the male Afghan population. Moreover, the majority of current literature among Afghan population in Iran, focused on the infectious diseases that could impose a risk on Iranian population such as HIV, tuberculosis, and malaria [9–11]. There are also a few studies in which the mental health and socioeconomic challenges of Afghan people have been explored in Iran [12, 13]. One study in Tehran province, showed a lower knowledge and poorer attitude, and practice among Afghan men compared to Iranian men in term of reproductive health; even though, the access was the same for both groups [14]. These findings highlight the importance roles of the context and culture in constructing the health behaviors of a specific population. These could also affect the health behavior of a women in seeking the maternity care. Therefore, we hypothesized that the behavior of Afghan women in seeking and utilizing of maternity care could be different in Iran with a different culture and environment.

The adequate maternity care is essential for a healthy pregnancy and favorable outcome [15]. However, despite the available prenatal care in Iran for all pregnant women, the evidence indicate higher mortality and perinatal complications among Afghan women and their offspring compared to the Iranian population [8, 16]. This is mainly due to the inadequate utilization of prenatal care in Afghan women. In other countries such as Canada, USA, and Switzerland; the lower utilization of maternity health services among refugees and asylum seekers has been attributed to a number of cultural and social factors such as stigma, language barrier, financial issue, gender- and cultural-sensitive issues [17–19]. However, in Iran as the second country with the highest number of Afghan refugees and immigrants in the world, there is no study that explored and addressed the issues in seeking maternity care and obstacles in having adequate access to such services among Afghan women. Thus, against this background, we explored the concerns of Afghan women in seeking prenatal care and described the potential obstacles toward adequate access to such services among pregnant Afghan women in Iran.

## Methods

### Study setting and sampling

This was an exploratory qualitative study which lasted from June 2019 to August 2019. Using Convenience sampling, the participants were recruited at three community health centers with highest Afghan visitors in south Tehran, in which the largest Afghan communities are located. The recruitment was continued until no novel information was produced during interviews (30 participants) and data saturation was achieved.

### Inclusion and exclusion criteria

We included the pregnant Afghan women aged 18–49 years old. The participants were approached by the interviewers. A brief introduction of the study objectives and the process of the interview were presented by the interviewers for all the women. After a verbal consent to participate, a written consent was obtained from the participant prior to the interview. Those who did not consent to participate, have any kind of physical disability, have not being permitted by accompanying family members, and were not pregnant at the time of interview were excluded. All the participants consented to attend the interview completed the study.

### Data collection

The sociodemographic information was collected using a researcher-developed structured questionnaire. The face-to-face interviews were conducted to explore the concerns of Afghan women in seeking prenatal care services and the potential obstacles toward adequate access to such services. An interview guide was used to conduct the interview. The interviewers were female Afghan graduate students at Tehran University of Medical Science and could speak Persian fluently. Although, language has been reported as a barrier in immigrant studies [19]; in present study, it was not an issue as the Persian is the first language of majority of Afghan people. Prior to the study, all the interviewers were trained and instructed about the objectives of the study and become familiar with the content of interview guide and the interview procedure. Informed verbal and written consents were obtained prior to the interview from all participants. The interviewer initiated the interview session by collecting the sociodemographic information and recording them in the designated sheet. The main interview session included asking open-ended questions which were explored more in detail by close-ended questions to achieve a comprehensive understanding of Afghan women’s concerns and experienced obstacles in seeking prenatal care. To ensure the participant’s privacy and convenience, the interviews took place in a separate room at corresponding community health center, in which only the participant and interviewer were present. Each interview approximately lasted an hour and was audio-taped with prior permission of participant. Additionally, the participants facial expression and side comments were noted during the interview. The interview was continued until no novel information was produced. Following the interview, the interviewers addressed the participants’ concerns related to the maternity and child health and a box of vitamin supplement was offered as a token for appreciation.

### Data analysis

All interviews were transcribed into the original language (Persian); and used for analysis. The thematic content analysis was employed to explore and analyze the data. To ensure the trustworthiness (validity) of data analysis, we followed the six stages of thematic analysis, described by Braun and Clarke [20]. The transcripts were read several times to extract the key codes (familiarization). Similar codes were identified (coding) and grouped as the subcategory and the main themes were created grouping the similar subcategories after the careful consideration and consensus between the researchers (generating theme). The MAXQD software version.10 was used to help with data analysis and extracting the codes, categories and themes. The themes were reviewed, named, and verified by other researchers (reviewing and naming the theme). The triangulation technique considering the opinions and comments of field experts at Tehran University of Medical Science and Kyoto University was used to ensure the credibility and dependability (reliability) of data analysis. In addition, the results were read and verified by participants who were available and could be reached to ensure the reliability of their statements (member check), and where necessary, their opinions and comments were accounted for in data analysis. The final results were translated into English by a bilingual researcher and further back-translated into Persian by another bilingual researcher to ensure the credibility and dependability of translated manuscript.

### Ethical consideration

The protocol of this study was reviewed and approved by the institutional review board (IRB) of both Kyoto University, Japan and Tehran University, Iran. Informed consent was obtained from all participants prior to the interview. They were assured regarding their information confidentiality. During the interview, pseudonyms were used to ensure participants’ anonymity and privacy.

## Results

### Demographic characteristics

Table 1 illustrates the participants’ characteristics. Thirty pregnant Afghan women aged 18–43 (mean age = 30.4) were interviewed in this study. Almost half of them were illiterate. Less than a quarter were employed. The length of stay in Iran, was at least 5 years for half of the participants. The majority of them were married to an Afghan man (86.7%), whereas, others had Iranian husband. Only 4 participants (13.3%) had insurance who were all legal migrants. Almost a third (9 persons) were illegal migrants.

**Table 1:** Participants’ characteristics

		n (%)
Age, mean (SD)	30.4 (6.90)	
Education	Illiterate	13 (43.3)
	Primary school	7 (23.3)
	Secondary school	8 (26.7)
	High school or higher	2 (6.7)
Legal status	Legal	21 (70)
	Illegal	9 (30)
Employment	Employed	7 (23.3)
	Unemployed	23 (76.6)
Husband nationality	Iranian	4 (13.3)
	Non-Iranian	26 (86.7)
Insurance	Have	4 (13.3)
	Do not have	26 (86.7)
Length of stay	Less than 5 years	16 (53.3)
	5 years or more	14 (46.7)
Number of children	No child	3 (10
	1	7 (23.3)
	2–4	18 (60)
	5 or more	2 (6.7)
Parity	Primipara	3 (10.0)
	2–3	16 (53.3)
	≥ 4	11 (36.7)
Total		30 (100)

### Determinants of access and barriers to prenatal care

#### Unaffordable diagnostic and screening tests

Most of the participants, especially those with illegal status, complained about financial struggles in accessing maternity care at some governmental and private hospitals. They preferred to visit community health centers as it is cheaper and insured people could access services for free. Almost all participants felt content about the free primary care at these centers and consider it a bonus for a healthy pregnancy.

*“I’d rather come here [community health center], it is much cheaper than private hospitals and clinics, my husband is a simple labor at a construction company, they don’t pay well to immigrants. Sometimes, I don’t even have the money to come here (40-45 years old, multipara, 12 years in Iran).”*

*“My husband and I are young and still struggling with financial issues; some of the services here are free for everyone, no matter where are you come from (18-24 years old, primipara, 2 years in Iran).”*

The majority of those with good economic status also preferred to receive the primary prenatal care at community health center as it is cheaper than governmental and private hospitals and clinics.

*“We have no financial issues; thanks to god. Though, I’d prefer to come here [community health center] to receive the prenatal care; it’s affordable (35-40 years old, multipara, 14 years in Iran).”*

Despite the free prenatal care for insured women at community health centers, the price for a prenatal visit at some governmental hospitals and private sector are much higher in Iran. Furthermore, diagnostic and screening tests, prenatal sonography and laboratory tests are not available at health centers. Pregnant women have to attend governmental hospitals or private clinics to receive these services. Almost all participants believed that these services are costly and they cannot afford them. Some women did not access prenatal sonography or laboratory tests as it was beyond their affordable budget.

*“You can’t do sonography here, I usually go to private clinic to do it and it’s expensive. Even in government hospital it’s expensive (35-40 years old, multipara, 14 years in Iran).”*

*“Once I had to do a sonography and some blood tests and since they don’t have them here [community health center] I went to a hospital and they charge me a lot! I won’t do it again! (18-24 years old, multipara, 2 years in Iran).”*

#### Ineligibility and inadequate coverage of health insurance

The public health insurance is now available for all the registered Afghans. However, 26 participants did have insurance and it appeared that almost half of the participants, as legal migrants, were not aware that they were entitled to health insurance.

*“I don’t have health insurance; I think that there is now an insurance for Afghans with visa (18-24 years old, multipara, 3 years in Iran).”*

*“I have none, I think Afghan people can’t have health insurance same as Iranian (18-24 years old, multipara, 8 years in Iran)”.*

On the other hand, 17 women were well-aware of health insurance for refugees, however, they could not or did not apply for it. One of the reasons was the illegal status, which disqualify them for application. However, 13 eligible women did not apply as they believed that the coverage of insurance is limited and does not worth the premium monthly bill. Although, there were four participants who had health insurance, complained about the lack of coverage for costly services such as prenatal sonography and screening tests.

*“We don’t have valid visa to stay in Iran [illegal immigrant] that’s why we cannot apply for insurance (30-35 years old, multipara, 6 years in Iran).”*

*“My husband recently learnt that Afghan migrant with a valid stay [legal immigrant] could apply for insurance, but we heard from some of our Afghan friends that’s it does not cover some services and procedures and you can only use it in government hospitals, so I think it does not help us a lot and not worth applying (35-40 years old, multipara, 10 years in Iran).”*

*“We recently apply for public health insurance, but wherever I go, they don’t accept it except here. It did not make any difference (30-35 years old, multipara, 8 years in Iran)”*

#### Transportation issues

*Seventeen* participants were living near the corresponding community health center and either walked or used public transport to get to the community health center. Some participants were brought by their husband using a private motorbike or car.

“*I walk; it takes only 10 minutes. Very close (40-45 years old, multipara, 12 years in Iran).”*

*“I usually take a taxi; it’s 5 minutes ride (30-35 years old, multipara, 2 years in Iran).”*

*“Usually, my husband brings me with his car (35-40 years old, multipara, 14 years in Iran).”*

*“My husband has a motorbike, we usually come here with that. However, I feel uncomfortable sometimes, I don’t think I can go for delivery on a motorbike (25-30 years old, multipara, 5 years in Iran).”*

Thirteen participants lived far away from the health center and, for these women, distance was one of the main obstacles for seeking maternity care. It caused some to miss appointments and there were also difficulties in finding a transport to the health center.

*“It is 20 minutes ride. There is no bus or cheap vehicle to reach here from our place. So I usually have to wait to come with my husband or son with our car, the problem is they are at work every day and sometimes I miss some appointments with my doctor (18-24 years old, multipara, 4 years in Iran).”*

*“We live in a village far from city, every time, it takes almost a day for me to come to the city and go back home, I usually come with minibus. I need to leaves early in the morning otherwise I will miss my doctor appointment. It happened many times (25-30 years old, multipara, 4 years in Iran).”*

#### Stigma and discrimination

Some women had bitter experiences or experienced discrimination. Some participants did not like to be questioned about their nationality because they perceived that as discrimination. However, it may be the tone or language used when being asked about their nationality which led to participant’s mistaken perception that they were being discriminated against.

*“Once I went to a private clinic and the secretory asked me whether I’m an Afghan in such repugnant way! I got a bit irritated but I didn’t say anything, I don’t like such questions when people ask me! I feel like they discriminate me because I’m an Afghan (30-35 years old, multipara, 6 years in Iran).”*

One participant was annoyed by criticisms made by doctor and midwife regarding her irresponsible behavior against her and her child’s health. Apparently, there was a misunderstanding. The woman was offended and felt discrimination. A few participants reported similar experiences.

*“Once I got insulted by a female doctor [midwife] because I had forgotten the exact date of my appointment. The doctor also got angry and shouted at me; she told me; why you Afghan people are so inconsiderate and careless about your health. In a way she was right but she did not have the right to call me by nationality, I felt insulted [irritated] (18-24 years old, primipara, 2 years in Iran).”*

*“It happens to everyone [to miss a maternity care], but we [Afghans] are also concern about our child’s health. I feel abandoned and isolated every time that my doctor argued me on a missing appointment just because I’m an immigrant doesn’t mean they can behave us like that [annoyed] (40-45 years old, multipara, 5 years in Iran).”*

Lack of knowledge and sensitive attitude obviously led to some of the mistaken perceptions among participants. For instance, a young participant was apparently upset as she was questioned by a doctor about the history of vaccination for hepatitis and rubella after she disclose her nationality. Apparently, she felt stigmatized by these questions being asked and did not realize that they were asked to all women.

*“Many Iranian people think because we are coming from a poor country we are carrying some kind of infectious disease like tuberculosis or hepatitis. In my last pregnancy, when I went to a hospital for delivery, after the female doctor realize that I’m an Afghan, she asked whether I received some kind of vaccine for a disease [don’t know the name], or whether I was tested for hepatitis or not. What do they really think? I don’t now, but we are not infectiousness [irritated] (18-24 years old, multipara, 4 years in Iran).”*

#### Waiting time

Although, there were many visitors at the community health centers during the interview time (9–11 am); a few participants complained about the waiting time.

*“It happens many times that you need to wait for long time because usually it crowded here. however, it’s like everywhere I think (40-45 years old, multipara, 5 years in Iran).”*

*“Somedays are crowded, however, they are very fast in visiting the patients; so often, I don’t need to wait long time. Especially in the early morning or late afternoon (25-30 years old, multipara, 5 years in Iran).”*

In addition, six participants who had maternity experiences in Afghanistan, considered the waiting time nothing compared to what they experienced in Afghanistan. However, compared to Iranian standard waiting time, they believed that the waiting time in community health centers and government hospitals is longer than private sector.

*“It’s nothing compared to the time that you need to wait in Afghanistan (35-40 years old, multipara, 5 years in Iran).”*

*“Here is much better, you don’t need to wait long like our hometown [in Afghanistan] (40-45 years old, multipara, 12 years in Iran).”*

#### Cultural concerns

The majority of statements were in line with the patriarchal culture of Afghan people. There were even some women who refused to attend the interview because their husband did not allow them. The disclosure of some information regarding the personal life were also contingent on the husband’s permission. It appeared that most of the Afghan women actually follow their husband’s or husband families’ opinions and instructions out of respect as a cultural norm rather than the fear.

*“My husband does not allow me to go anywhere alone, I need to listen to my husband and respect his opinion, when he say; I can go, I will go (18-24 years old, multipara, 3 years in Iran).”*

*“I’m living with my husband family like many other Afghan women, the important decision about our daily life are usually taken by my husband and his family. My brothers and mother in law are in charge when my husband is away and I need to listen to them, if they don’t let me to go somewhere I don’t have to go, I respect them (18-24 years old, multipara, 3 years in Iran).”*

Gender sensitive issues were also mentioned as an obstacle in seeking maternity care. In strict Islamic culture, the direct touch of female body by a man other than husband is forbidden. Therefore, absence of a female doctor in health center, sometimes, led to refusing to be seen by a male doctor and thus a delay in accessing maternity care. This was even reported by educated Afghan women.

*“My husband’s family are very well educated and open-minded. However, once my husband denied to let a male doctor to examine me when he wanted to do sonography to check my baby health. I had to go to a female doctor after that, Though, I like my husband in that way. (25-30 years old, multipara, 5 years in Iran).”*

*“My husband didn’t want me to be visited by a male doctor when I had a back pain in my trimester; however, after my pain got worse, he took me to a private clinic whose doctor was female (18-24 years old, multipara, 3 years in Iran).”*

There were a few difficult experiences where mostly the husband’s family member such as mother or brother in law were against the routine maternity care and had even forbidden and refused to take the troubled pregnant women to the hospital. For example, a 32-year-old woman described her experience;

*“My mother in law does not believe in western medicine, she always complains about how western medicine spoiled our culture and traditional medicine. Once she didn’t even let my sister in law to go to hospital when she did not feel well in her fourth pregnancy because she believed that doctors will prescribe dangerous drugs and pills and it will hurt her baby (30-35 years old, multipara, 8 years in Iran).”*

Another unusual experience of a 34-year-old women was as below;

*“Once when my husband was far away for work, my brother in law did not allow me to go to doctor when I had bleeding during my pregnancy. He just moved from Afghanistan to here, he is a very strict Muslim and does not believe in medicine and doctors, he said everything is according to the god’s will and if he wants the baby dead then let it be! However, after I called my husband, he convinced my mother in law to take me to the doctor without letting him [brother in law] to know. It was fearful experience for me (30-35 years old, multipara, 6 years in Iran).”*

Most of these bitter experiences emerged from woman’s lack of autonomy originated from the man-dominant culture and values of Afghan society. In both above-mentioned scenarios, the husband and his family were at low socioeconomic status with low literacy. In addition, the second scenario occurred in an illegal-stay family.

#### Legal and immigration issues

The fear of deportation was the main concern to seek prenatal care for those who were either illegal refugees or their visa expired at the time of interview. Twenty-one participants in this study had valid immigration documents and seemed not to be worried about this issue as much as those of illegal-stay status. On contrary, the illegal Afghan women expressed the fear of visiting the health centers, particularly the government health facilities as they believed that they might be arrested and deported to Afghanistan. It appeared that these issues usually pushed them to visit the private health facilities where the expenses are high and they often cannot afford it. However, it appeared that the problem mainly stands for labour and birth cares. Where the identity documents of parents are required to create a health record and issue a birth certificate for the baby.

*“I didn’t go to hospital during my first pregnancy because my and my husband’s visas were expired and I feared if I go to hospital they will arrest us and send us back to Afghanistan (18-24 years old, multipara, 8 years in Iran).”*

*“For health services before delivery; here [community health center] it’s ok, because they don’t ask for identity documents, but for delivery, you need to go to hospital. Of course a private one, where they don’t care about identity documents [talk cautiously] (30-35 years old, multipara, 6 years in Iran).”*

*“I have to be careful in going around because I don’t want to cause problem for my husband, if they [authorities] know they will deport us. Here [community health center] is ok! But once I went to a hospital and they ask me for identity document to be admitted (30-35 years old, multipara, 7 years in Iran).”*

On the other hand, almost all the legal Afghan women appeared to be familiar with their rights for maternity care and childbirth as a legal resident and seemed to be satisfied with delivered services.

*“It’s not an issue for me, because we have valid visa and immigration documents that they need to create a record for admission, it’s not a problem for legal migrants in Iran. we have the same right as Iranian (30-35 years old, multipara, 7 years in Iran).”*

Four women who were married to Iranian men mentioned no problem in seeking health care in Iran. One of these women said;

*“My husband is Iranian and I’m not worried about these kind of immigration issues, I have health insurance and my husband support me. I’m happy [smile] (40-45 years old, multipara, 5 years in Iran).”*

There was interestingly an illegal refugee who seemed to be satisfied and had no concern about legal issues;

*“I was brought here by my Iranian friend who is also pregnant. Doctors and nurses don’t ask about our visa here, they are so kind, they just care about our health (18-24 years old, multipara, 4 years in Iran).”*

## Discussion

The present study investigated the potential barriers and concerns of pregnant Afghan women in utilization of prenatal care in Iran. The most frequent reported issues were financial struggles in seeking prenatal care at some governmental hospitals and private sector. The lack of capital for expensive prenatal procedures such as prenatal sonography and screening tests was another main obstacle in receiving adequate prenatal care. The cost of these procedures is usually high even at the government facilities and often beyond the affordable budget of Afghan people. The situation is even worse for undocumented Afghans as they usually visit the private facilities in the fear of arrest and deportation at the government facilities [21].

Lack of health insurance is one of the most important obstacles in seeking health services among immigrants and refugees; particularly for the illegal ones [18]. In Iran; since 2016, the public health insurance had become available for all registered Afghans. Nowadays, the insured Afghans could benefit from the same health services covered by the public health insurance as the Iranian citizen [21]. However, it appeared that the majority of participants in our study did not have health insurance. Apart from the illegal status of some participants that did not allow them to apply for the health insurance, inadequate coverage of some prenatal services such as screening and diagnostic sonography and laboratory tests was the main source of disappointment in applying for pubic health insurance. The public health insurance is funded by the government; however, the reimbursement and service coverage are inappropriate; therefore, most of the Iranians use private insurance companies with better coverage and reimbursement. Unfortunately, Afghan refugees and immigrants are not eligible to apply for such insurance scheme. We recommend negotiation and collaboration between international agencies and engaged parties in order to promote the coverage of existent insurance for refugees and immigrants in Iran.

The difficulties in transportation was another important issue raised by some participants. The financial constrains complicate the situation for those living in remote areas as the transportation cost appeared to be high and even unaffordable in some cases. This issue was mainly raised by the participants of illegal status; whose socioeconomic status was low and they were living in remote areas. This could affect the maternal and child health of such vulnerable population by limiting their access to appropriate and affordable health care [22].

The waiting time seemed to be short at the community health centers, in where we conducted the interviews; however, some participants reported longer waiting times, especially at government hospitals; particularly, in seeking services such as sonography. These findings were in line with the evidence from other developing countries; the weak infrastructure of health system and insufficient health professionals in some remote areas; particularly in government sector, reported to be the main reasons for such long waiting hours in most developing countries [23]. Likewise, in Iran. this issue still exists in some remote areas; however, in big cities such as Tehran, it is not a problem anymore.

Evidence has shown that the perceived discrimination; feeling of abandonment, and isolation among immigrants and refugees could reflect themselves in person’s perceived quality of life and satisfaction in host society [24, 25]. Similarly, in present study, some participants reported bitter experiences in which they felt discriminated and stigmatized, mainly due to their nationality. Similar experiences were reported among Afghan people living in Iran [26]. This is mainly attributed to the lack of the intercultural-competency of health personnel. The language barrier could also lead to miscommunication and such bitter experiences of abandonment and isolation [27]; however, in Iran, it is not an issue as the Afghan people could fluently speak and communicate in Persian. Thus, we emphasized the necessity of interventions to enhance the intercultural-competency of health personnel delivering health services at Afghan-concentrated communities in Iran.

The fear of deportation was another important obstacle in seeking prenatal care especially at government sector among refugees of present study. It appeared that registered Afghan have enough knowledge of their rights as a legal resident and did not express such fear in seeking health care. In fact, this issue was mainly reported among undocumented migrants and refugees in surveys from other countries [28–30]. It could limit the access to the appropriate health care among illegal migrants and refugees. Moreover, the alternative practices which are often available at private sector, are usually beyond the affordable budget of this poor population and this could lead to higher morbidity and disability among them [18]. Therefore, expanding the safety net services for illegal migrants and refugees in Iran, at least for pregnant women, should be a priority of health authorities to ensure a safe and healthy pregnancy and guarantee the health of offspring in this vulnerable population.

### Limitations

There were some pitfalls and drawbacks in present study. First, it is a general knowledge that the small sample size and the sampling procedure in qualitative research limit the representativeness of the results. Moreover, the hardships in reaching pregnant Afghan women willing to participate in the study reduced our ability in recruiting the women of different background, especially the women of low socioeconomic status, illegal-status and those belong to the strict Muslim families whom were reluctant or forbidden by husband to attend the interview. However, we believe that recruiting female Afghan interviewers facilitated the communication during the interview, enrich the content, and enhance the extent of knowledge to which we expected to reach in this study. Second, missing the views of women who choose not or did not have access to prenatal care could cause bias, this occurred as the detecting and reaching this hide population is really hard in Iran.

## Conclusion

This is the first study in Iran that explored the concerns and barriers to prenatal care among pregnant Afghan women. The findings emphasized the necessity of available and most importantly, affordable prenatal care for Afghan women in Iran. Providing an affordable health insurance with adequate coverage of prenatal care services, could reduce the financial burden, facilitate the access to prenatal care, and ensure the health of mothers and offspring among this vulnerable population. Apparently, the situation for illegal Afghan migrants and refugees is more critical and should be considered by international agencies and regional health authorities where this population predominantly reside. The issues of fear and concern of deportation, at least, must be removed for the illegal Afghan mothers to ensure the safety and optimal health of both mother and child. Further studies are of critical necessity to explore the issues surfaced themselves in present study and to determine the prevalence and distribution of such issues and further find solutions to address them in future national policy and guidelines.

## Data Availability

The data analyzed in the current study are not publicly available to protect the privacy and anonymity of the participants.

## Abbreviations

IRB: Institutional review board
FDG: Focused group discussion
